# Chip Pad Inspection Method Based on an Improved YOLOv5 Algorithm

**DOI:** 10.3390/s22176685

**Published:** 2022-09-04

**Authors:** Jiangjie Xu, Yanli Zou, Yufei Tan, Zichun Yu

**Affiliations:** 1 School of Electronic and Information Engineering, Guangxi Normal University, Guilin 541000, China; 2 Robot Laboratory, Guangxi Normal University, Guilin 541000, China

**Keywords:** OCAM, attention, YOLOv5, chip pads, artificial intelligence

## Abstract

Chip pad inspection is of great practical importance for chip alignment inspection and correction. It is one of the key technologies for automated chip inspection in semiconductor manufacturing. When applying deep learning methods for chip pad inspection, the main problem to be solved is how to ensure the accuracy of small target pad detection and, at the same time, achieve a lightweight inspection model. The attention mechanism is widely used to improve the accuracy of small target detection by finding the attention region of the network. However, conventional attention mechanisms capture feature information locally, which makes it difficult to effectively improve the detection efficiency of small targets from complex backgrounds in target detection tasks. In this paper, an OCAM (Object Convolution Attention Module) attention module is proposed to build long-range dependencies between channel features and position features by constructing feature contextual relationships to enhance the correlation between features. By adding the OCAM attention module to the feature extraction layer of the YOLOv5 network, the detection performance of chip pads is effectively improved. In addition, a design guideline for the attention layer is proposed in the paper. The attention layer is adjusted by network scaling to avoid network characterization bottlenecks, balance network parameters, and network detection performance, and reduce the hardware device requirements for the improved YOLOv5 network in practical scenarios. Extensive experiments on chip pad datasets, VOC datasets, and COCO datasets show that the approach in this paper is more general and superior to several state-of-the-art methods.

## 1. Introduction

Chip pad inspection is the database for wafer alignment inspection and alignment calibration, and it is one of the key technologies in the field of semiconductor manufacturing automation. It ensures a high level of real time and accuracy in chip alignment inspection and calibration and plays an important role in the subsequent decisions of chip alignment inspection and calibration technology [1,2].

In the actual wafer manufacturing process, chip alignment greatly affects the yield of wafer chips. In recent years, with the rapid development of deep learning, deep learning has provided a new solution for chip alignment inspection technology, which can quickly detect pads and pad defects [3,4,5], and indirectly detect and correct chip alignment by detecting chip pads. In practical alignment work, the chip solder joints are detected by a target detection algorithm, and if the machine detects a chip solder joint, the chip pads are considered to be unaligned, and if no chip pads are detected during the inspection process, the alignment is considered to be successful. Most of the state-of-the-art target detection algorithms use convolutional neural networks, which can meet the requirements of high real-time and high accuracy in industry and have achieved better results in most of the target detection tasks, as opposed to the traditional manual detection or region proposal detection method [6].

At present, the two most mainstream directions in object detection algorithms are the two-stage detection algorithm and the single-stage detection algorithm. The classical algorithms of two-stage object detection methods include Faster R-CNN [7] and R-FCN [8]; although the R-CNN series algorithms have high accuracy, they cannot achieve the requirement of real-time detection due to a large number of network parameters and slow detection speed. Among the single-stage object detection algorithms are the SSD [9] and YOLO [10,11,12,13] series algorithms; YOLOv5 [14] is by far the best algorithm to achieve accuracy and speed, and it achieves a good balance of detection accuracy and detection speed; therefore, this paper uses the YOLOv5 network as a baseline network for chip pad inspection. However, when the network is directly applied to the chip pad inspection task, satisfactory inspection results are not achieved.

In chip pad inspection tasks, the small target size of chip pads makes it difficult for conventional detection models to obtain chip pad targets from complex backgrounds effectively. Conventional CNNs use a large number of parameters and FLOPs to achieve better detection performance; however, mobile devices with limited memory and computational resources (smartphones and virtual machines) cannot use large networks for deployment and inference. Therefore, the ideal chip pad detection model needs to meet at least two requirements for efficient small target detection performance and being lightweight.

Attentional mechanisms are widely used to solve small target detection problems. Numerous research works have shown that attention plays a very important role in human perception [15]. When viewing a picture, people usually do not pay attention to the whole picture but focus their attention on a particular part of it. Similarly, in machine learning, one can better capture the visual structure of object features by using local sequence features to depict the whole object [16]. SE (Squeeze-and-Excitation networks) [17] uses 2D global pooling to compute channel information, which enhances the channel feature information, and a large number of experiments have demonstrated that the SE module can well solve the “what” problem in computer vision tasks, but it is missing the position information. Bottleneck Attention Module (BAM) [18] enhances the position information to some extent by using spatial relations, but it cannot depict well the position relations of the target in the target detection task. The later CA (Coordinate attention) [19] well represents the position relationship of the target object by compressing the X, Y information, but it lacks the channel information. The CBAM (Convolutional block attention module) [20], on the other hand, uses channel attention mechanism and spatial attention mechanism to solve the problem of “what” and “where” from two dimensions and improves both information features at the same time. However, these attentions acquire the channel features or spatial features from local, lacking the contextual relationship between features, and cannot acquire the long-range dependencies between feature information.

In existing work on object detection, the introduction of the attention mechanism layer has been effective in improving detection, but most of the literature does not explain the particularities of the attention mechanism layer design [13,21,22,23,24]. A large number of workers in the field of NAS (Neural Architecture Search) have demonstrated through extensive experiments that there are principles to follow in the design of neural networks, the utility of which is speculative, and that serious deviations from these principles often lead to a deterioration in network quality, and that fixing these deviations promotes architectural improvements. Kaiming et al. [25] proposed residual learning, which solves the problem of deep network degradation while deepening the network, forms a thin and long network, and greatly improves the network efficiency. Zagoruyko et al. [26] proposed a method to increase the width of the convolutional layers, and the use of larger convolutional kernels improved the expressiveness of the network to some extent. After that, more lightweight models were proposed. Howard et al. [27,28] balanced the width and resolution of the network by introducing a modulation factor and experimentally demonstrated that the network efficiency reached higher after the two parameters were balanced. Tan et al. [29] proposed a new scaling method, which uses a simple but efficient composite coefficient to uniformly scale all dimensions of depth, width, and resolution and achieves the optimal balance of the network through scaling. Han et al. [30] further investigated the expression bottleneck problem in single-layer design, identified the single-layer design law, and proposed Rexnet, which was further improved on the existing one.

To solve the chip pad inspection problem, this paper proposes an improved YOLOv5 network algorithm, which not only ensures the improvement of small target chip pad inspection accuracy but also reduces the model parameters and network computation to meet the industrial production automation requirements.

The main contributions of our work are as follows:

- A new attention module is proposed. Through a nested structure, the long-range dependencies between different feature information are constructed pioneeringly to strengthen the correlation between features and reduce the information loss from shallow to high-level information, effectively improving the detection effect of small target chip pads detection task.
- A design guideline for a single attention layer is proposed. Influenced by the design of neural network architectures, we conduct an in-depth study of the network expressiveness of a single attention layer and propose a design guideline by which the network expressiveness of the attention layer can be effectively improved.
- The existing YOLOv5 network has been improved. The parameters and detection effects have been balanced to achieve better overall network performance and lighter weight.

## 2. Materials and Methods

### 2.1. Datasets

**Figure 1:**
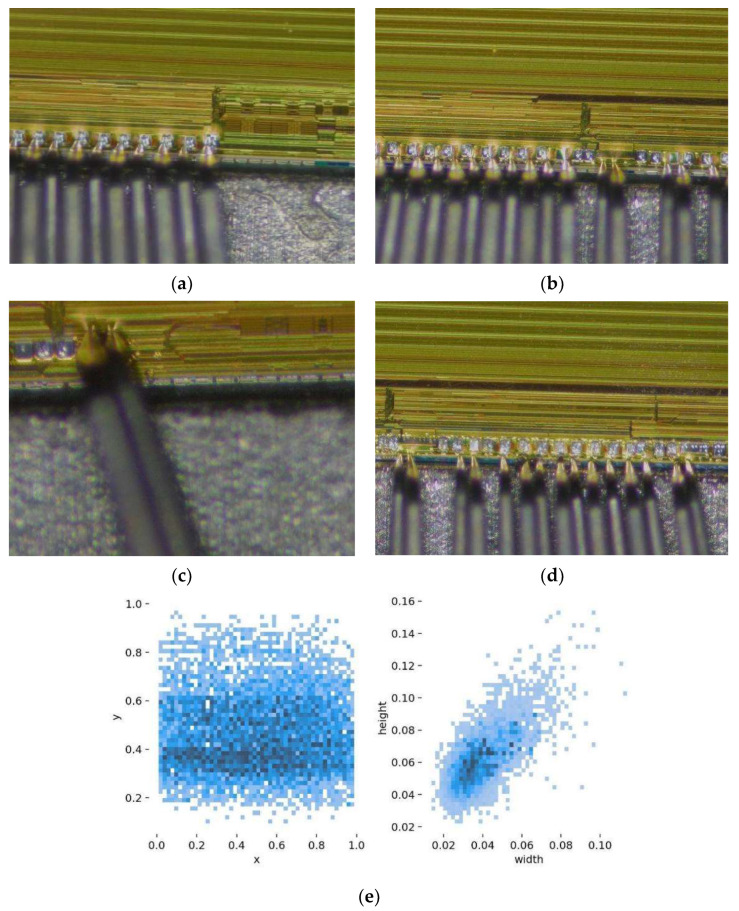
Wafer Solder Joint Dataset. (**a**,**b**) Sample image contains chip pad A; (**c**) Sample image contains chip pad B; (**d**) Sample image contains chip pad A and chip pad B; (**e**) Target frame distribution.

The chip pads dataset was obtained from a semiconductor company in Guangxi, China. With reference to the actual chip pads in the inspection, we used an industrial camera with 6112 × 3440 pixel resolution for image acquisition under different lighting conditions to construct an image dataset of chip pads; the industrial camera contains a 10 cm focal length and 200× magnification eyepiece and a 2000 w pixel CMOS sensor and some samples of the dataset are shown in Figure 1a–d where chip pad A (rig) is the chip pad where the probe needs to be aligned in the inspection, and chip pad B (wro) is the chip pad where the probe does not need to be aligned in the inspection. The acquired data are filtered and organized, and some of the images are cropped and data enhanced to enhance the usability of the samples. Then the labeling software is used to label the different chip pad image samples from the collected images. The chip pad dataset is constructed with 6000 chip pad A and 2200 chip pad B. The total of 1464 image samples are divided into training and test sets by 9:1. Figure 1e visualizes the distribution of target box size and the distribution of target box appearance position in the dataset. The two figures show that the target box size is uneven, the number of small targets is large, and the target appearance position is concentrated in the middle of the pictures.

### 2.2. Improvements to the YOLOv5 Network

YOLOv5 (5.0) contains four models, namely YOLOv5s, YOLOV5m, YOLOv5l, and YOLOv5x, of which YOLOv5s is the smallest model in the YOLOv5 series, with a size of about 14 M, more suitable for industrial production applications.

The framework of YOLOv5 can be divided into four parts, namely, input, feature extraction, backbone, and prediction layers. The input part feeds the images into the network in the form of three channels; the feature extraction layer mainly consists of C3 modules which perform feature extraction by Darknet53; in the backbone part, the multiscale aggregation of features of different sizes is performed by the feature pyramid structure (FPN), and finally, in the prediction layer the network performs target detection and network prediction output.

In this paper, an OCAM attention module is introduced in the feature extraction layer of YOLOv5 to improve the efficiency of small target detection in the target detection network. Referring to the attention mechanism layer design guidelines proposed in this paper, an OCAM layer is embedded behind each C3 module in the feature extraction layer, and then the network is scaled with reference to α×β≈2 (α is the module depth and β is the expansion ratio), and the number of convolution kernels in each layer is compressed, with the output of the first layer module being 32, the output of the second layer module being 64, the output of the third layer module being 128, and the fourth layer module The output of the first layer module is 32, the second layer module is 64, the third layer module is 128, the fourth layer module is 256, and finally the aggregation is performed at the backbone layer.

**Figure 2:**
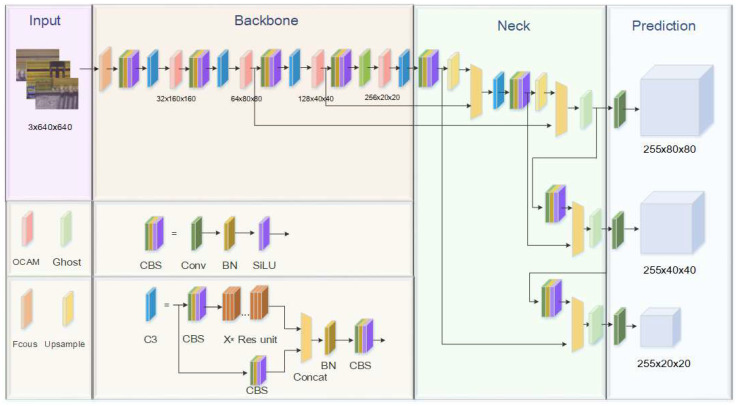
Proposed improved YOLOv5s network structure diagram.

In the backbone layer, inspired by the literature [31,32,33,34,35], we use the Ghost module instead of the original convolution module in this paper to generate more redundant features using less computation and parameters, which makes the network lighter and more suitable for industrial automation production while ensuring that the network accuracy is not affected. In the prediction layer, we use CIOU [36] instead of the original GIOU [37]. Compared with the GIOU loss, the CIOU loss takes into account the overlapping area of the bounding box, the distance to the centroid, and the consistency of the bounding box aspect ratio, which is more consistent with the actual detection work. The structure of the improved YOLOv5s network is shown in Figure 2.

### 2.3. OCAM Attention Module

#### 2.3.1. OCAM Contextual Relationship Construction

In this section, the paper first reviews some of the work on constructing contextual links and then extends this section by constructing long-range dependencies between different features.

Long-range dependencies: Non-Local Network [38] proposed a pioneering approach for capturing long-range dependencies by aggregating the global context of a particular query to each query location. GC-Net [39] further simplifies such relationships and unifies them into a three-step generic framework for global context modeling with the following formulation:

(1) $$z_{i}=F(x_{i},\delta (\overset{N_{p}}{\underset{j=1}{\sum }}\alpha_{j}x_{j}))$$

$\sum_{j=1}^{N_{p}}\alpha_{j}\chi_{j}$ denotes the global context features obtained, j denotes the index on the feature map, $\delta \left(\;\right)$ denotes the feature transform that captures the channel correlation, and$\;F\left(\;\right)\;$denotes the feature fusion function that aggregates the global context features to each location.

**Figure 3:**
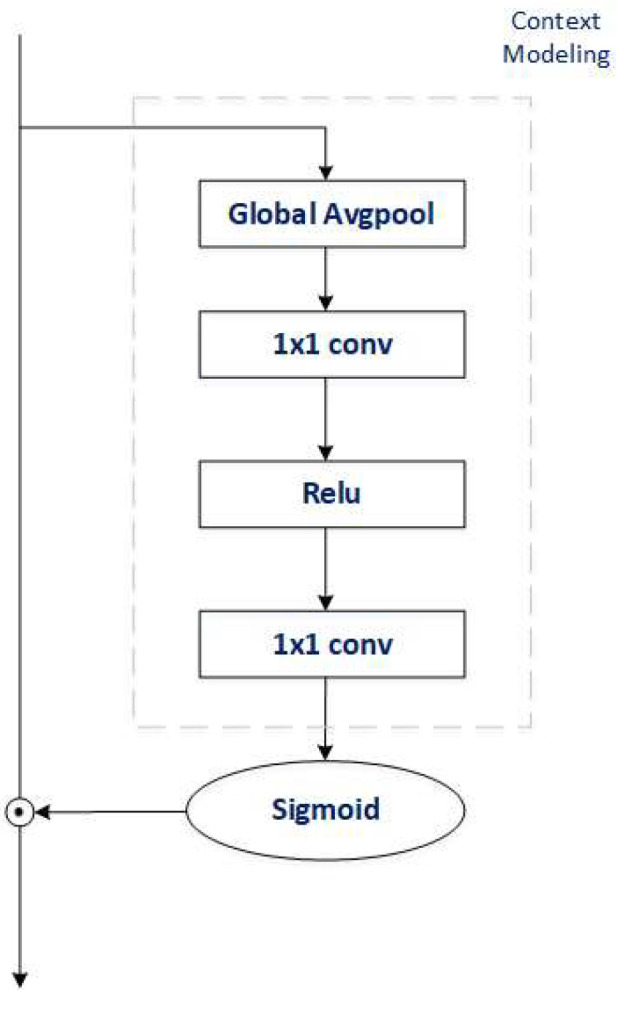
Attentional Simplification Chart.

Attentional characteristics: Here, we simplify the existing channel and spatial attention information; as in Figure 3, the attention features can be reduced to a series of operations, such as compression and fully connected layers, to obtain feature information and then feature excitation by sigmoid. Thus, we generalize the two operations:

(2) $$\delta (\overset{N_{p}}{\underset{j=1}{\sum }}M_{c}\left(x_{j}\right))$$

(3) $$\delta (\overset{N_{p}}{\underset{j=1}{\sum }}M_{p}\left(x_{j}\right))$$

where *j* denotes the index on the feature map, $\sum_{j=1}^{N_{p}}M_{c}\left(\;\right)$ denotes a series of transformations including convolution, compression, and expansion channels to obtain the channel features, $\sum_{j=1}^{N_{p}}M_{p}\left(\;\right)$ denotes a series of transformations, including convolution, compression, and expansion channels, to obtain the position features, and $\delta \left(\;\right)$ denotes the activation function of the feature map.

By transforming Equation (1) and embedding (2) (3) into (1), we can obtain a single feature information relationship containing contextual relationships:

(4) $$z_{c}=F(x_{i},\delta (\overset{N_{p}}{\underset{j=1}{\sum }}M_{c}\left(x_{j}\right))$$

(5) $$z_{p}=F(x_{i},\delta (\overset{N_{p}}{\underset{j=1}{\sum }}M_{p}\left(x_{j}\right))$$

Feature relationship fusion: The $F\left(\;\right)$ in (1) is extended. $F\left(\;\right)$ is a feature mapping function that aggregates the above features with the below information in a linear transformation, and in this paper, the below information is extended to a mapping containing the location feature information:

(6) $$F\left(\;\right)=\delta (\overset{N_{p}}{\underset{j=1}{\sum }}M_{p}\left(\chi_{j}\right))$$

Thus, synthesizing Equations (1)–(6), a long-range dependency is constructed between channel feature information and position features to strengthen the correlation between different features, which is described by Equation:

(7) $$Z_{i}=\overset{N_{p}}{\underset{j=1}{\sum }}M_{p}(x_{i},\delta (\overset{N_{p}}{\underset{j=1}{\sum }}M_{c}\left(x_{j}\right)))$$

From this relationship, the OCAM attention module is proposed in this paper.

#### 2.3.2. OCAM Attention Module Structure

**Figure 4:**
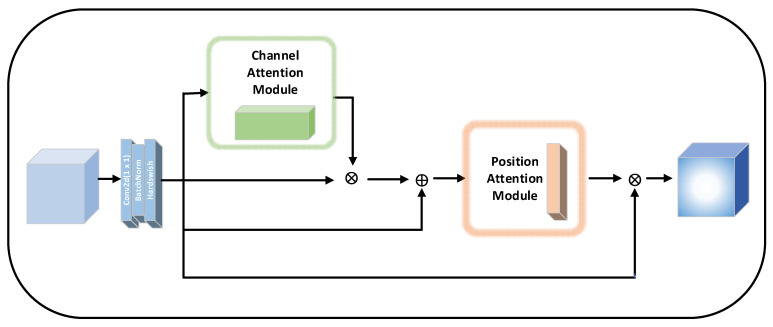
Structure of OCAM attention module.

As shown in Figure 4, the proposed OCAM attention module in this paper consists of three parts. Firstly, a filter module consisting of 1×1 conv, batchnorm, and hardswish functions is used to reduce the computational effort by reducing the data dimensionality for different levels of information. Then a four-branches branching structure is entered. In the structure, for the specificity of the target detection task, the position attention mechanism is introduced to deal with the position information, and the channel attention mechanism is used to deal with the color features. Based on the above relationships, the channel attention module and the position attention module are constructed and using feature superposition between the two attention modules, long-range dependencies are established to strengthen the correlation between the features and enhance the information of the two features, and then and the input feature maps are aggregated. Finally, the internal residual structure is introduced. The connection between the shallow feature information and the deep information is ensured, and the information loss is reduced.

As can be seen from Figure 4, for a given input feature map $F\in R^{C\times H\times W}$, OCAM sequentially outputs a 3D filter feature map $M\in R^{C\times H\times W}$, a 1D channel feature map $M_{c}\in R^{C}$, and two 2D location feature maps $M_{PA}\in R^{C\times H}\&R^{C\times W}$. The overall attention process can be summarized as follows:

(8) $$F^{\prime }=M\left(F\right)$$

(9) $$F^{\prime \prime }=F+F⨂M_{c}\left(F^{\prime }\right)$$

(10) $$F^{\prime \prime \prime }=F⨂M_{PA}\left(F^{\prime \prime }\right)$$

##### Channel Attention Module

**Figure 5:**
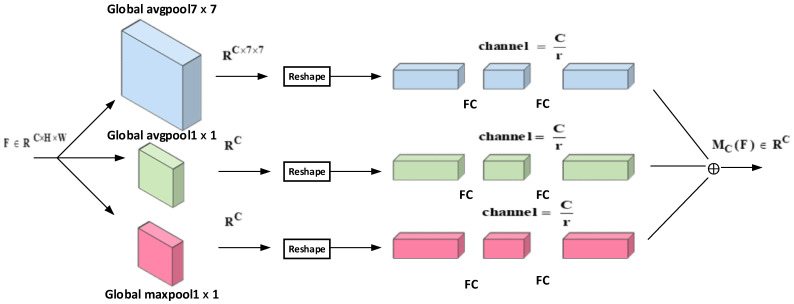
Channel attention of OCAM attention module.

As shown in Figure 5, the channel features of each layer contain different channel responses, each response corresponding to a category. In the target detection task, the detection frame requires different structural information for different sizes of targets. It is usually desired to reflect more structural and channel information in the channel feature extraction to improve the robustness of the network for different target detection. Therefore, in this paper, three pooling methods, 7×7 global average pooling, 1×1 global average pooling, and 1×1 global maximum pooling, are used to compress the channel features from different structures to enhance the structural information among channels. To facilitate the feature mapping in the subsequently aggregated channels, the pooled feature maps are reshaped to obtain a unified feature mapping, and a multilayer perceptron (MLP) is used for each layer to enhance the cross-channel information from different structures, where r is the compression rate. In summary, the overall structure of the channel attention mechanism in this paper is as follows:

(11) $$\begin{array}{c} X_{1}=X\times (\sigma (MLP\left(Resize\left(AvgPool\left(X\right)\right)\right)+\sigma (\mathrm{MLP}\left(Resize\left(\mathrm{MaxPool}7\left(X\right)\right)\right)\\ +\sigma \left(\mathrm{MLP}\left(Resize\left(\mathrm{MaxPool}\left(X\right)\right)\right)\right) \end{array}$$

where $X_{1}$ denotes the output feature map, X denotes the input feature map, $AvgPool\left(\;\right)$ denotes 1×1 global average pooling, $AvgPool7\left(\;\right)\;$denotes 7×7 global average pooling, $MaxPool\left(\;\right)$ denotes 1×1 global maximum pooling, $Resize\left(\;\right)\;$performs normalization operation on the feature map, $MLP\left(\;\right)$ denotes multilayer perceptron, r is the compression rate, and σ denotes the activation function.

##### Position Attention Module

Inspired by Coordinate attention [19], the precise position information is captured and encoded from both X, Y directions and encoded to form a direction-aware and position-sensitive attentional feature map to be applied to the input feature map in a complementary way, thus enhancing the position feature information.

**Figure 6:**
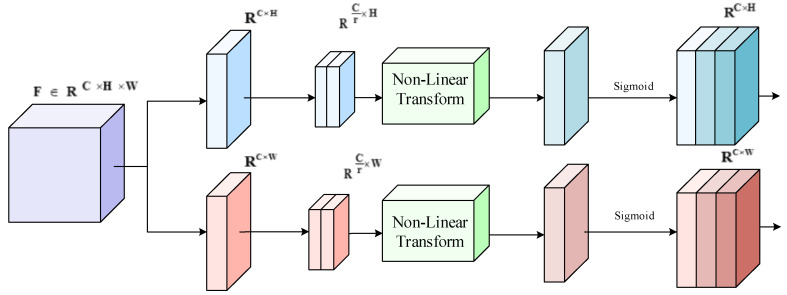
Position attention module structure diagram.

The structure of the position attention module is shown in Figure 6. To implement the coordinate information embedding, two one-dimensional feature encoding vectors are obtained by first decomposing the horizontal and vertical directions, respectively, through global average pooling. Then, the coordinate information of different dimensions is mapped by nonlinearity. Finally, the two feature maps containing X and Y coordinates are output as follows:

(12) $$X_{1}=Conv(\mathrm{Nonlinear}\left(\mathrm{Batch}\left(Conv\left(\mathrm{AvgpoolH}\left(X\right)\right)\right)\right)$$

(13) $$X_{2}=Conv(Nonlinear\left(Batch\left(Conv\left(AvgpoolW\left(X\right)\right)\right)\right)$$

where X is the input feature map, $X_{1}$ and $X_{2}$ are the output feature maps, $Nonlinear\left(\;\right)$ is the nonlinear activation function, $AvgpoolH\left(\;\right)$, $AvgpoolW\left(\;\right)\;$are the intermediate feature maps encoding the spatial information in horizontal and vertical directions, $Batch\left(\;\right)\;$is the batchnorm operation, $Conv\left(\;\right)\;$is the 1×1 convolution operation, σ is the sigmoid activation function, and r is the compression rate.

### 2.4. Attention Layer Design Guide

This section analyses the theoretical aspects of how to design an attention mechanism layer to improve the expressiveness of the network as much as possible, in terms of both position and channel scaling dimensions, and gives an experimental analysis in Section 3.

Position: In NLP works, the literature [40,41] pointed out that the low-rank output of SoftMax is unable to express the complexity of high-rank space, and a network SoftMax representation bottleneck occurs. The authors proposed a method to enhance the representativeness by processing the output layer to improve the network representation problem and improve the accuracy of the model. Is there the same representation problem in CV work for a single layer? The literature [30] theoretically demonstrates that in CV work, the same single-layer representation bottleneck exists, and through extensive experiments, it is found that guiding the number of design layer channels by monotonically increasing the number of layer orders can effectively improve the network representation and make the network achieve higher accuracy. This implies that the expressiveness of the network can still be effectively changed by handling the position of a single layer in CV work. Based on the above study, we speculate that the network representativeness of a single attention layer is location-dependent, and studying it may provide a more efficient choice of attention layer locations.

Channel ratio: A large number of network architectures [42,43] have shown that deeper networks can capture more features, wider networks can capture more fine grains and pixels, and any single dimension can improve the expressive power of the network, but this improvement is not the most efficient way to improve, as, in most language modeling, the network can only reach the best gain once the width and depth reach some balance. Efficientnet [29] demonstrated that from a single dimension, the network gains are very limited when the network reaches very deep or very wide networks. The authors proposed a new composite scaling method that uses the composite coefficients φ to perform a principled and uniform scaling of the width, depth, and resolution of the network, which effectively enhances the network expressiveness by compound scaling of width, depth, and resolution. Based on the above work, we speculate that the same composite scaling approach also exists in the design of a single attention layer, and studying it may provide a more efficient scaling of the attention layer.

Design Method: Through the experimental results in Section 3, considering the expressiveness of the target detection network and avoiding the representation bottleneck, this paper proposes a design guideline for attention layers: (1) designing more attention layers in the Backbone layer can enhance the network expressiveness. (2) The scaling of the attention layers should satisfy α×β≈2. In this way, the network representation bottleneck can be avoided and the network efficiency can be improved.

### 2.5. Training Details

During the experiments, the image size was fixed at 640 × 640 input, the initial value of the learning rate was 0.01, the learning rate was reduced by using the cosine annealing strategy, the number of iterations was 500, the batchsize was 32 in the chip pad dataset, Microsoft COCO [44] and PASCAL 2007 + 2014 dataset are 64.

The experiments are done on Linux version 5.4.0-96-generic, Ubuntu 7.5.0 with Intel(R) Core(TM) i7-12700K CPU and NVIDIA (Santa Clara, CA, USA) GeForce RTX 3090 GPU with 24 G video memory size, and The GPU is accelerated using Cuda 10.2.

## 3. Results

In this section, using the chip pads dataset, the attention layer design approach is first experimentally analyzed, then the performance of the proposed method is verified, and finally, the improved YOLOv5 network is evaluated.

### 3.1. Experimental Analysis of Attention Level Design Guide

The YOLOv5s (5.0) version and the OCAM attention module proposed in this paper are used as the basis. The experimental dataset selected is the chip pads dataset. Two variable factors are introduced to regulate the depth and width of the network; the variable factor $\alpha \in \left[1,\;4\right]$ is taken as an integer and indicates the number of layers of the designed attention mechanism, which is usually described as the depth of the layer in the design of neural networks. $\beta \in \left(0,\;3\right]$ indicates the ratio of the number of channel changes (β = number of output channels/number of input channels); when β>1, the layer is an extended layer and when β<1, the layer is a compressed layer, so that it is more convenient to discuss the effect of different channel change ratio on the network, where the value of β can be theoretically arbitrary, through a large number of experiments, when β doubles, the parameters increase by $\beta^{2}$. Considering that in the case of sacrificing a large number of parameters, if the accuracy does not reach a good gain, it will lead to too low network efficiency, so β is taken as (0, 3] to achieve a higher network efficiency in a limited range.

Inspired by the literature [45,46,47], the attention layer was designed in the target detector behind the C3 feature extraction module in the YOLOv5s network. Based on the above analysis, the study is specified into two aspects:

(1) Measure the network expressiveness when attention layers are set at different stages (Use mAP@0.5 as an indicator).
(2) Two variable factors α, β are introduced. α and β are scaled with β=1 as the baseline, and the effects of different α and β modulations on the network performance (Use mAP@0.5 as an indicator) are investigated to find the optimal scaling ratio of the attention layer.

#### 3.1.1. Study of the Embedding Position in the Attention Layer

**Figure 7:**
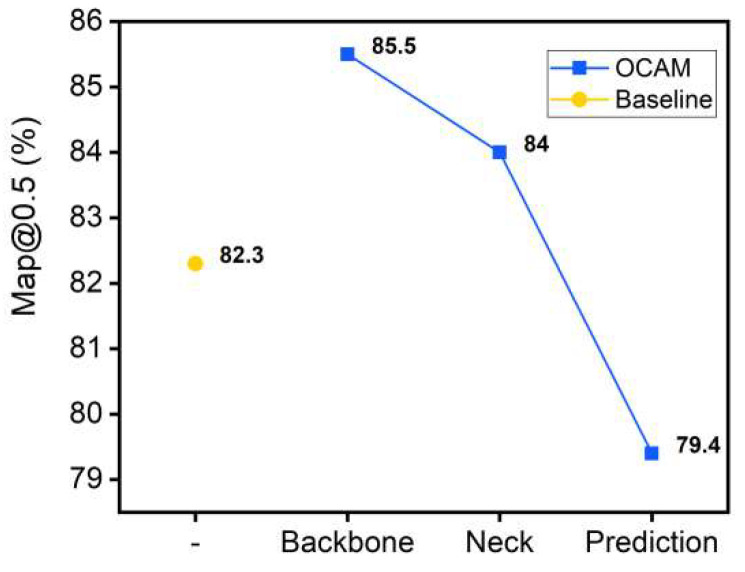
Network accuracy when designing the same OCAM layer in different layers.

Figure 7 shows the network performance in Baseline (YOLOv5s) when the same attention layer is embedded in the three phases. It is observed that the baseline accuracy is 82.3%, and the network performance is affected when the attention layers are designed in different positions. The network performance is enhanced at the Backbone and Neck layers, and the network accuracy is improved. However, this performance decreases sharply with the depth of the network, which impairs the efficiency of the network, and the network shows a negative gain when the attention layer is embedded in the Prediction layer. As the network goes deeper, the attention layer appears as a representation bottleneck in the network, which affects the efficiency of the attention layer. The experiments show that this representation bottleneck is less affected when the attention layer is designed in shallow networks, and the network accuracy is relatively higher.

#### 3.1.2. Study of Channel Scaling in the Attentional Layer

**Figure 8:**
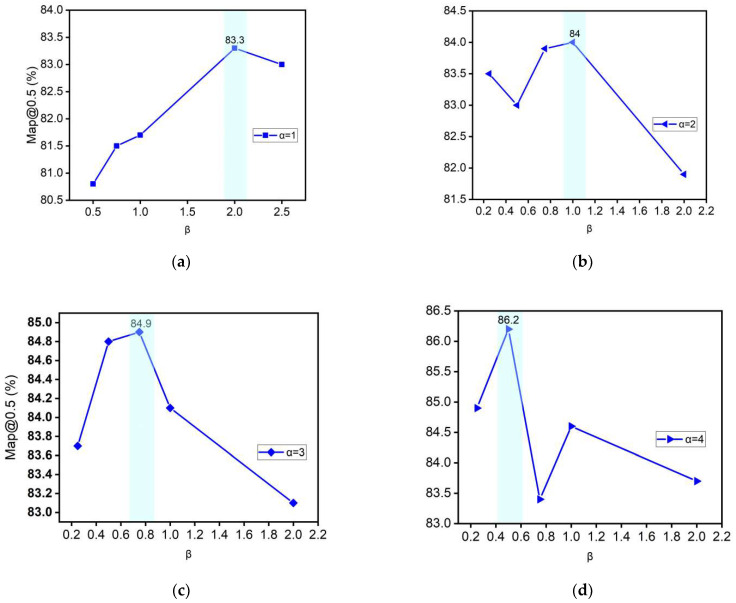
(**a**–**d**) shows the adjustment of the attention layer using different depth (α) and scaling (β ). Find the effect of scaling β on accuracy at different α with β=1 as the baseline, respectively, for the current α.

Figure 8 represents the study of the width and depth of the attention layer at the Backbone layer. By setting the attention layer in Backbone with different depths (indicated by α), two modulation factors were introduced to regulate the depth and channel ratio of a single layer, which can be obtained from the experimental results: (1) At the same depth, different channel ratios have an impact on the network performance, ignoring the large fluctuating values. As the channel ratio increases, the overall network accuracy at different channel ratios shows a mountain shape, which may be due to the network scaling benefit, where the network performance is optimal and the accuracy peaks when the depth and width are balanced. (2) With the increase in α, the expressiveness of the network gradually increases and the highest accuracy that the network can achieve rises, while the highest accuracy can reach 86.2% and the deeper attention layer can effectively improve the network accuracy. (3) With the increasing of $\alpha \in \left[1,4\right]$, the location of the peak also changes, β peaks near 2 for α=1, β peaks near 1 for α=2, β peaks near 0.75 for α=3, and β peaks near 0.5 for α=4.

#### 3.1.3. Experimental Conclusions about the Attention Layer

(1) The target detection network has an expression bottleneck at different positions of the attention layer, and this effect is minimized at the backbone layer, and in addition, the depth of the attention layer can help enhance the network performance.
(2) Based on Efficientnet [29], we find that the performance of the attention layer has a regular pattern; summarizing the above experiments, we get that the network performance reaches the peak when α×β≈2.

### 3.2. Results and Analysis of Chip Pad Targets Detection

The improved YOLOv5 model of this paper was compared with the mainstream target detection model using several metrics, including model size, parameters, floating point operations per second (FLOPs), and average accuracy (mAP@0.5), to evaluate the model performance, as shown in Table 1.

First, it can be seen that the average accuracy of the improved model in this paper is 87.5%, which is 4.9% better than the original YOLOv5s(5.0) algorithm, 3.5% better than the latest YOLOv5s(6.0) algorithm, and 2.5% better than the latest YOLO series algorithm YOLOX [48], and in terms of average accuracy, the algorithm in this paper is better than most of the existing algorithms. In terms of the accuracy of a single chip pad, the recognition accuracy of chip pad A is 89.8%, which is higher than other algorithms, and the recognition accuracy of chip pad B is 85.2%, which is slightly higher than the 84% of YOLOv5s(5.0), which shows that our detection accuracy and robustness to different targets are greatly improved. Secondly, the improved model size is 10.8 M, which is easy to be deployed in real industrial applications. Finally, the improved model parameters are 5.5 M and FLOPs are 15.6 G, which are slightly higher than the current optimal algorithms YOLOv5-lite-g [49] and Efficientdet-d0. In summary, the improved method in this paper has higher detection accuracy for small target chip pads, the model is more lightweight, and it achieves a better balance between accuracy and parameters, which is more suitable for deployment in practical applications. It is more suitable for deployment in practical applications.

**Figure 9:**
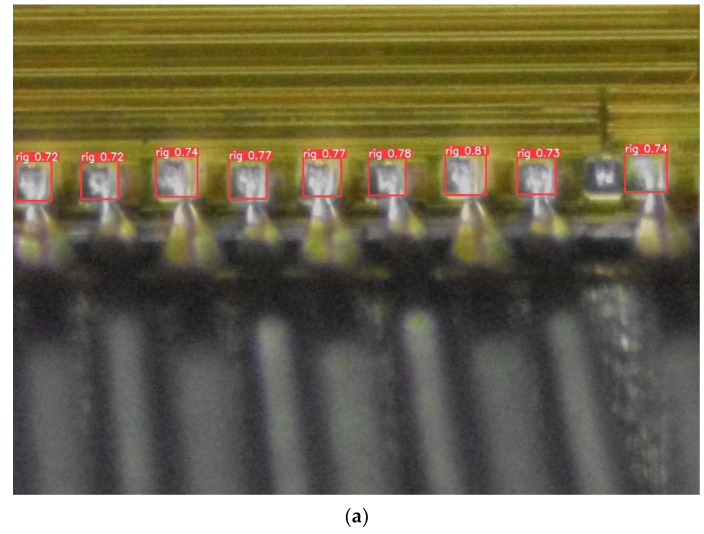

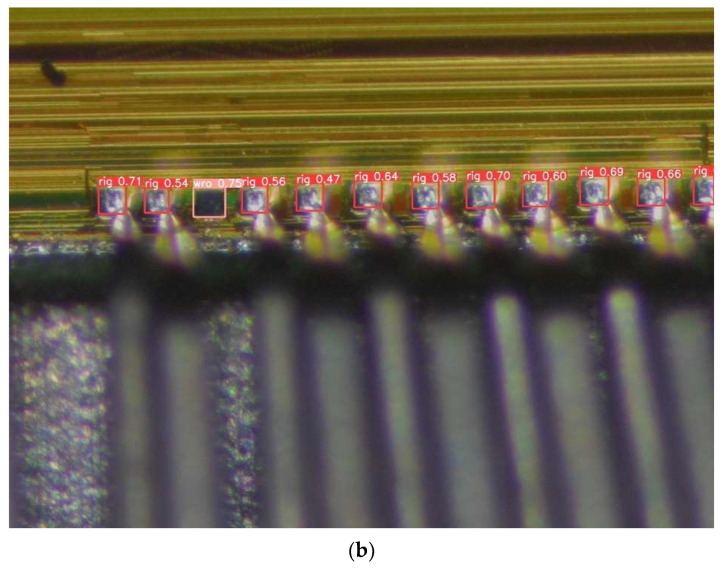
(**a**,**b**) Our method.

**Figure 10:**
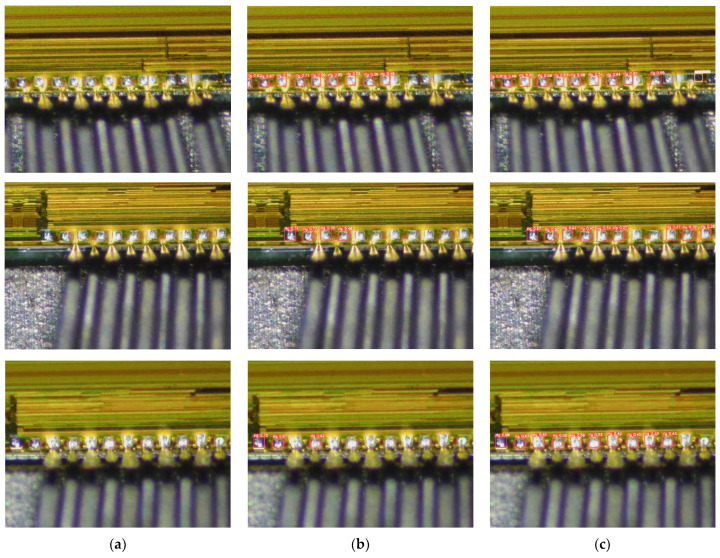
Comparison between our method and YOLOv5s model on-chip pad dataset. (**a**) Origin. (**b**) YOLOv5s. (**c**) Our.

The actual detection of chip pads is shown in Figure 9 and Figure 10. As can be seen from the figure, compared to the original YOLOv5s, this algorithm not only detects more chip pads but also detects chip pads with larger masking areas. In addition, the detection rate of the improved model in this paper is higher than the original algorithm in the detection of the same chip pads. This means that, in practice, our detection results have better robustness and detection success rate.

**Figure 11:**
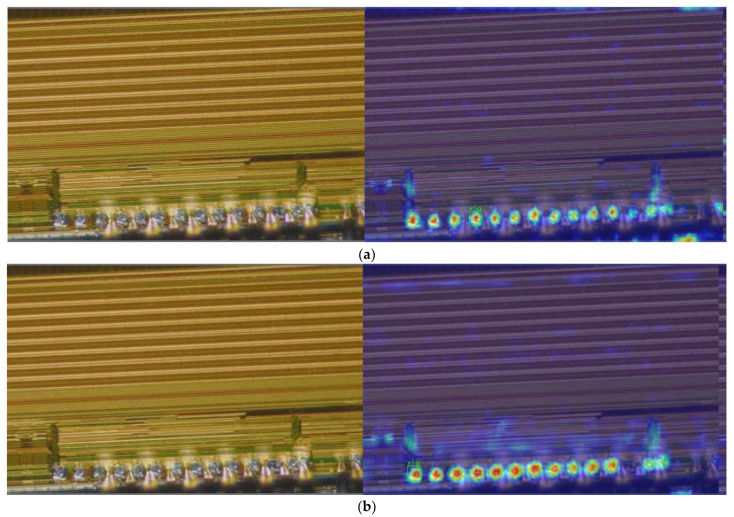
Grad-CAM visualization results. (**a**) YOLOv5s. (**b**) Our method.

To further illustrate the inspection results, the paper visualizes the results using Grad-CAM [50]. As shown in Figure 11, the red part of the figure represents the degree of aggregation of features by the network, and the darker the color of the region indicates that the network pays more attention to the features in that region during the detection process, and vice versa, the region is ignored. From the figure, it can be seen that during pad detection, the method in this paper extracts more features, and the network pays more attention to the features of different targets. From the visualization results, it can be seen that the method in this paper can effectively enhance the features of small target pads during detection.

## 4. Discussion

### 4.1. Attention Module Experiment

Table 2 compares the attentional approach proposed in this paper with some of the mainstream attentional approaches, selecting average accuracy (Map@0.5) as a reference indicator. From the experimental results, the average accuracy (Map@0.5) of the original baseline reaches 82.6%, the accuracy of Chip pad A is 85%, and the accuracy of Chip pad B is 80.24%. After adding the attention method of this paper, the accuracy reaches 86.5%, 88.8% for Chip pad A and 84.2% for Chip pad B, which are higher than some mainstream attention methods. Therefore, the method in this paper is more suitable for small target detection tasks and can achieve greater accuracy gains in the target detection work.

### 4.2. Experiment on Design Scheme of Attention Layer

Table 3 compares the design scheme proposed in this paper with the conventional design scheme. As can be seen from the table, the average accuracy of the original YOLOv5s reaches 82.6%, with a model size of 13.7 M, parameters of 7.0 M, and FLOPs of 16.5 G. After adding OCAM, Map@0.5 improves to 85.6%, up 3%, with a slight increase in parameters. After network tuning according to the design guidelines in this paper, the network characterization bottleneck is reduced, and the accuracy is further improved to 86.5%, up 0.9%, while the network parameters are decreased, which is an impressive optimization, which means that the overall network performance is improved by redesigning the network structure and the network efficiency is effectively improved.

### 4.3. Ablation Experiment

To demonstrate the performance of the proposed method more visually, we conducted ablation tests, and the results are shown in Table 4.

From the results, the original accuracy of YOLOv5s is 82.6%, the model size is 13.7 M, parameters are 7.1 M, and flops are 16.5 G. When the Ghost module is added, the accuracy remains almost the same, but the model size, parameters, and computation are reduced, which makes the network lighter. After adding the OCAM module, the accuracy increases to 86.5% and Map@0.5 increases by 3.9%, while the network parameters remain almost the same. Finally, all the methods are added to the YOLOv5s network and the accuracy reaches 87.5%, while the model size, parameters, and FLOPs are reduced compared to the original YOLOv5s. This means that the proposed network has a good balance of accuracy and parameters, and the improved network requires less training speed and equipment, which ensures that the method can be put into practical use more easily.

### 4.4. VOC and COCO

To further verify the effectiveness of the improved YOLOv5 model, the improved YOLOv5 model of this paper was compared with the mainstream target detection model on the PASCAL VOC2007+2012 dataset and Microsoft COCO, and the comparison results are shown in Table 5 and Table 6.

First, from Table 5, we can see that the average accuracy of this model is 82.8% on the VOC dataset, which is 3.4% better than the original YOLOv5s(5.0) algorithm, 2.5% better than the latest YOLOv5s(6.0) algorithm, and 0.8% better than the YOLOv3 model, which is an effective improvement in detection accuracy. Finally, the improved model has 5.5 M parameters and FLOPS are 15.6 G, which is slightly higher than YOLOv5-Lite-g and Efficientdet-d0. The overall network shows better performance on the VOC dataset with fewer parameters and networks. The overall network shows better performance on the VOC dataset, with fewer parameters and network computation to achieve better detection results.

As can be seen from Table 6, the average accuracy of the improved model in this paper is 58.0% on the COCO dataset, which is 2.6% better than the original YOLOv5s(5.0) and 1.2% better than the latest YOLOv5s(6.0) algorithm. The accuracy is higher than other algorithms in the table. The improved model has a size of 10.9 M, parameters of 5.5 M, and FLOPS of 15.6 G, which is slightly higher than some lightweight models, but the overall performance is slightly better than the current optimal model.

In summary, the improved YOLOv5 network shows good performance on different datasets, with effective improvement in detection accuracy and good robustness to different detection targets, which proves the effectiveness of the improved method in this paper.

## 5. Conclusions

In this paper, we propose an improved YOLOv5 chip pad detection network, which has better performance than the existing single-stage detector. The improved model mainly introduces the attention layer OCAM, which effectively enhances the correlation between different features, improves the long-range dependencies between features, and improves the detection performance of small target chip pads. The attention layer design scheme proposed in this paper can effectively improve the network expression capability of the attention layer and improve detection efficiency. The experimental results show that the improved network can achieve better detection results with fewer parameters, with a Map@0.5 improvement of 4.9% on the wafer chip pads dataset and equally effective on the COCO and VOC datasets. The improved YOLOv5 network has a lighter network model and lower hardware requirements, making it more suitable for practical industrial applications.

The improved YOLOv5 model proposed in this paper achieves better performance than most existing target detection models, but there is still some room for improvement in terms of detection speed and detection efficiency. On the other hand, an ideal industrial detection model needs to have the ability to work under different working scenarios; however, the pad datasets used for the training of the model in this paper were collected under sufficient lighting conditions with rich information on pad color features, for scenarios with poor lighting conditions, the lighting conditions result in color features and pad location features not being obvious, and the pad features cannot be extracted effectively during detection. Therefore, this method is not suitable for extreme lighting conditions. If the above shortcomings can be effectively addressed, the method in this paper can improve the self-monitoring performance of the wafer measurement die machine to a certain extent and achieve automatic correction of alignment anomalies, which can greatly reduce the labor cost in the industry, and at the same time, due to the enhanced lightweight of the model, the cost of the embedded equipment in the actual deployment work can be reduced, so the method in this paper has good industrial application value. In future work, researchers can focus on more complex work scenarios to improve the efficiency of the pad detection model in poorly lit scenarios where color features are not obvious to improve the robustness of industrial inspection.

## Figures and Tables

**Table 1:** Performance comparison of our method with mainstream target detection models on chip pad dataset.

Method	Model	Parameters	FLOPs	AP@0.5		mAP@0.5
				Solder Joint A	Solder Joint B	
Efficientdet-d0	15 M	3.7 M	2.50 B	0.69	0.48	0.586
SSD-mobile	118 M	25.06 M	29.2 G	0.67	0.43	0.55
YOLOv3	117 M	61.5 M	154.9 G	0.821	0.742	0.781
YOLOX-S	34.3 M	9.1 M	27.03 G	0.87	0.83	0.85
YOLOv5-Lite-g	10.7 M	5.4 M	15.6 G	0.799	0.764	0.782
YOLOv5s-5.0	13.7 M	7.1 M	16.5 G	0.813	0.84	0.826
YOLOv5s-6.0	14.4 M	7.0 M	15.9 G	0.878	0.808	0.84
Our	10.8 M	5.5 M	15.6 G	**0.898**	**0.852**	**0.875**

**Table 2:** Attention module comparison.

Method	AP@0.5		mAP@0.5
	Chip Pads A	Chip Pads B	
**YOLOv5s (Baseline)**	0.85	0.802	0.826
+CBAM	0.867	0.794	0.831
+CoordAtt	0.86	0.814	0.837
+SE	0.871	0.767	0.819
+ECA	0.883	0.777	0.83
+Our	**0.888**	**0.842**	**0.865**

**Table 3:** Comparison of attention layer design solutions.

Method	Model	Parameters	FLOPs	mAP@0.5
YOLOv5s	13.7 M	7.0 M	16.5 G	0.826
+OCAM (normal)	13.8 M	7.1 M	16.7 G	0.856
+OCAM (guide)	13.5 M	6.3 M	16.3 G	**0.865**

**Table 4:** Ablation experiments.

Method	Model	Parameters	FLOPs	mAP@0.5
YOLOv5s	13.7 M	7.1 M	16.5 G	0.826
YOLOv5 + Ghost	12.8 M	6.2 M	14.9 G	0.825
YOLOv5 + OCAM	13.5 M	6.3 M	16.3 G	0.865
Our	10.8 M	5.5 M	15.6 G	0.875

**Table 5:** Performance comparison on VOC2007+2012 dataset.

Method	Model	Parameters	FLOPs	mAP@0.5
Efficientdet-d0	15 M	3.75 M	2.50 B	0.78
SSD-mobile	118 M	25.06 M	29.2 G	0.77
YOLOv3	117 M	61.5 M	155.2 G	0.82
YOLOX-S	34.3 M	9.1 M	27.03 G	0.75
YOLOv5-Lite-g	10.7 M	5.5 M	15.6 G	0.811
YOLOv5s-5.0	13.8 M	7.1 M	16.5 G	0.794
YOLOv5s-6.0	14.4 M	7.0 M	16.0 G	0.803
Our	10.9 M	5.5 M	15.6 G	**0.828**

**Table 6:** Performance comparison on COCO dataset.

Method	Model	Parameters	FLOPs	mAP@0.5
YOLOv3-tiny	23.0 M	6.06 M	6.96 B	33.1
YOLOv4-tiny	33.7 M	8.86 M	5.62 G	40.2
Efficientdet-d0	15.0 M	3.75 M	2.60 B	52.2
YOLOv5-Lite-g	10.7 M	5.5 M	15.6 G	57.6
YOLOv5s-5.0	14.0 M	7.3 M	17.0 G	55.4
YOLOv5s-6.0	14.0 M	7.23 M	16.0 G	56.8
Our	10.9 M	5.5 M	15.6 G	**58.0**

## Data Availability

The data presented in this study are available on request from the corresponding author.

## References

[1] Kim H., Lee K., Jeon B., Song C. (2009). Quick wafer alignment using feedforward neural networks. IEEE Trans. Autom. Sci. Eng..

[2] Smith R., Collins S. (1989). A wafer-to-wafer alignment technique. Sens. Actuators.

[3] Li H., Hao K., Wei B., Tang X.S., Hu Q. (2022). A reliable solder joint inspection method based on a light-weight point cloud network and modulated loss. Neurocomputing.

[4] Peng Y., Yan Y., Chen G., Feng B. (2022). Automatic CCM Solder Joint Inspection Method Based on Machine Vision. Meas. Sci. Technol..

[5] Hu Q., Hao K., Wei B., Li H. (2022). An efficient solder joint defects method for 3D point clouds with double-flow region attention network. Adv. Eng. Inform..

[6] Divvala S.K., Hoiem D., Hays J.H., Efros A.A., Hebert M. An empirical study of context in object detection. Proceedings of the 2009 IEEE Conference on Computer Vision and Pattern Recognition.

[7] Ren S., He K., Girshick R., Sun J. (2016). Faster R-CNN: Towards Real-Time Object Detection with Region Proposal Networks. IEEE Trans. Pattern Anal. Mach. Intell..

[8] Dai J., Li Y., He K., Sun J. (2016). R-fcn: Object detection via regionbased fully convolutional networks. Adv. Neural Inf. Process. Syst..

[9] Liu W., Anguelov D., Erhan D., Szegedy C., Reed S., Fu C.-Y., Berg A.C. (2016). Ssd: Single shot multibox detector. European Conference on Computer Vision.

[10] Redmon J., Farhadi A. Yolo9000: Better, faster, stronger. Proceedings of the IEEE Conference on Computer Vision and Pattern Recognition.

[11] Redmon J., Divvala S., Girshick R., Farhadi A. You only look once: Unified, real-time object detection. Proceedings of the IEEE Conference on Computer Vision and Pattern Recognition.

[12] Bochkovskiy A., Wang C.-Y., Liao H.-Y.M. (2020). Yolov4: Optimal speed and accuracy of object detection. arXiv.

[13] Yu Z., Shen Y., Shen C. (2020). A real-time detection approach for bridge cracks based on YOLOv4-FPM. Autom. Constr..

[14] Ultralytics, Yolov5. https://github.com/ultralytics/yolov5.

[15] Larochelle H., Hinton G.E. (2010). Learning to combine foveal glimpses with a third-order boltzmann machine. Adv. Neural Inf. Process. Syst..

[16] Corbetta M., Shulman G.L. (2002). Control of goal-directed and stimulus-driven attention in the brain. Nat. Rev. Neurosci..

[17] Hu J., Shen L., Sun G. Squeeze-and-excitation networks. Proceedings of the IEEE Conference on Computer Vision and Pattern Recognition, Salt Lake City.

[18] Park J., Woo S., Lee J.-Y., Kweon I.S. (2018). Bam: Bottleneck attention module. arXiv.

[19] Hou Q., Zhou D., Feng J. Coordinate attention for efficient mobile network design. Proceedings of the IEEE/CVF Conference on Computer Vision and Pattern Recognition.

[20] Woo S., Park J., Lee J.-Y., Kweon I.S. Cbam: Convolutional block attention module. Proceedings of the European Conference on Computer Vision (ECCV).

[21] Zhu X., Lyu S., Wang X., Zhao Q. TPH-YOLOv5: Improved YOLOv5 Based on Transformer Prediction Head for Object Detection on Drone-captured Scenarios. Proceedings of the IEEE/CVF International Conference on Computer Vision (ICCV) Workshops.

[22] Zhu L., Geng X., Li Z., Liu C. (2021). Improving YOLOv5 with Attention Mechanism for Detecting Boulders from Planetary Images. Remote Sens..

[23] Xu X., Zhang X., Zhang T. (2022). Lite-yolov5: A lightweight deep learning detector for on-board ship detection in large-scene sentinel-1 sar images. Remote Sens..

[24] Jiang Z., Zhao L., Li S., Jia Y. (2020). Real-time object detection method based on improved yolov4-tiny. arXiv.

[25] He K., Zhang X., Ren S., Sun J. Deep residual learning for image recognition. Proceedings of the IEEE Conference on Computer Vision and Pattern Recognition.

[26] Zagoruyko S., Komodakis N. (2016). Wide residual networks. arXiv.

[27] Howard A.G., Zhu M., Chen B., Kalenichenko D., Wang W., Weyand T., Andreetto M., Adam H. (2017). Mobilenets: Efficient convolutional neural networks for mobile vision applications. arXiv.

[28] Sandler M., Howard A., Zhu M., Zhmoginov A., Chen L.-C. Mobilenetv2: Inverted residuals and linear bottlenecks. Proceedings of the IEEE Conference on Computer Vision and Pattern Recognition, Salt Lake City.

[29] Tan M., Le Q. Efficientnet: Rethinking model scaling for convolutional neural networks. Proceedings of the International Conference on Machine Learning.

[30] Han D., Yun S., Heo B., Yoo Y. Rethinking channel dimensions for efficient model design. Proceedings of the IEEE/CVF Conference on Computer Vision and Pattern Recognition.

[31] Han K., Wang Y., Tian Q., Guo J., Xu C., Xu C. Ghostnet: More features from cheap operations. Proceedings of the IEEE/CVF Conference on Computer Vision and Pattern Recognition.

[32] Yan B., Fan P., Lei X., Liu Z., Yang F. (2021). A real-time apple targets detection method for picking robot based on improved YOLOv5. Remote Sens..

[33] Yu Y., Zhao J., Gong Q., Huang C., Zheng G., Ma J. (2021). Real-time underwater maritime object detection in side-scan sonar images based on transformer-YOLOv5. Remote Sens..

[34] Wan J., Chen B., Yu Y. (2021). Polyp Detection from Colorectum Images by Using Attentive YOLOv5. Diagnostics.

[35] Guo Z., Wang C., Yang G., Huang Z., Li G. (2022). MSFT-YOLO: Improved YOLOv5 Based on Transformer for Detecting Defects of Steel Surface. Sensors.

[36] Zheng Z., Wang P., Liu W., Li J., Ye R., Ren D. (2020). Distance-iou loss: Faster and better learning for bounding box regression. Proc. AAAI Conf. Artif. Intell..

[37] Rezatofighi H., Tsoi N., Gwak J., Sadeghian A., Reid I., Savarese S. Generalized intersection over union: A metric and a loss for bounding box regression. Proceedings of the IEEE/CVF Conference on Computer Vision and Pattern Recognition.

[38] Wang X., Girshick R., Gupta A., He K. Non-local neural networks. Proceedings of the IEEE Conference on Computer Vision and Pattern Recognition, Salt Lake City.

[39] Cao Y., Xu J., Lin S., Wei F., Hu H. Gcnet: Non-local networks meet squeeze-excitation networks and beyond. Proceedings of the IEEE/CVF International Conference on Computer Vision Workshops.

[40] Kanai S., Fujiwara Y., Yamanaka Y., Adachi S. (2018). Sigsoftmax: Reanalysis of the SoftMax bottleneck. Adv. Neural Inf. Process. Syst..

[41] Ganea O., Gelly S., B’ecigneul G., Severyn A. Breaking the SoftMax bottleneck via learnable monotonic pointwise nonlinearities. Proceedings of the International Conference on Machine Learning.

[42] Zoph B., Le Q.V. (2016). Neural architecture search with reinforcement learning. arXiv.

[43] Sharir O., Shashua A. (2017). On the expressive power of overlapping architectures of deep learning. arXiv.

[44] Lin T.-Y., Maire M., Belongie S., Hays J., Perona P., Ramanan D., Doll´ar P., Zitnick C.L. (2014). Microsoft coco: Common objects in context. European Conference on Computer Vision.

[45] Zhang Z., Zhang X., Peng C., Xue X., Sun J. Exfuse: Enhancing feature fusion for semantic segmentation. Proceedings of the European Conference on Computer Vision (ECCV).

[46] Chaib S., Liu H., Gu Y., Yao H. (2017). Deep feature fusion for vhr remote sensing scene classification. IEEE Trans. Geosci. Remote Sens..

[47] Ghiasi G., Lin T.-Y., Le Q.V. Nas-fpn: Learning scalable feature pyramid architecture for object detection. Proceedings of the IEEE/CVF Conference on Computer Vision and Pattern Recognition.

[48] Ge Z., Liu S., Wang F., Li Z., Sun J. (2021). Yolox: Exceeding yolo series in 2021. arXiv.

[49] Yolov5-lite. https://github.com/ppogg/YOLOv5-Lite.

[50] Selvaraju R.R., Cogswell M., Das A., Vedantam R., Parikh D., Batra D. Grad-CAM: Visual Explanations from Deep Networks via Gradient-Based Localization. Proceedings of the 2017 IEEE International Conference on Computer Vision (ICCV).

